# A blind area of family planning services in China: unintended pregnancy among unmarried graduate students

**DOI:** 10.1186/1471-2458-13-198

**Published:** 2013-03-06

**Authors:** Yuanzhong Zhou, Chengliang Xiong, Jinwen Xiong, Xuejun Shang, Guohui Liu, Meimei Zhang, Pin Yin

**Affiliations:** 1 School of public health, Zunyi medical college, Dalian Road 201, Zunyi 563000, China; 2 Institute of Family Planning, Tongji Medical College, Huazhong University of Science and Technology, Hangkong Road 13, Wuhan 430030, China; 3 Department of O&G, Hubei Provincial Corps Hospital, Chinese People′s Armed Police Forces, Minzhu Road 475, Wuhan 430061, China; 4 Department of Andrology, Nanjing General Hospital of Nanjing Military Region, Zhongshan East Road 305, Nanjing 210002, China; 5 Institute for Population and Family Planning of Chongqing City, Honghuang Road 18, Chongqing 400020, China; 6 Sexual Health Education Research Center, Capital Normal University, You An Men Xitoutiao 10, Beijing 100069, China; 7 Department of Epidemiology, School of Public Health, Tongji Medical College of Huazhong University of Science and Technology, Hangkong Road 13, Wuhan 400030, China

**Keywords:** Education, Graduate, Contraception, Unplanned pregnancy, Universities, Family planning services, China

## Abstract

**Background:**

Status of premarital sex, unintended pregnancy and associated factors among Chinese graduate students remain unclear. And unmarried graduate students’ needs of family planning services seem to be ignored. In the present study, we ascertained the prevalence rate of premarital sex and unintended pregnancy, as well as estimated the possible factors associated with unintended pregnancy among unmarried Chinese graduate students, and evaluated their reproductive health needs.

**Methods:**

We obtained the representative sample of graduate students using a multistage, stratified, cluster design, and collected data using a questionnaire.

**Results:**

We obtained 11936 responders. Premarital sexual intercourse was acknowledged by 24.2% of responders; unintended pregnancy was acknowledged by 4.8% of responders (19.8% of students active in premarital sex); and abortion was acknowledged by 4.6% of responders (96.7% of pregnant students). In multivariate analysis, the identified risk factors for unintended pregnancy among both genders that were active in premarital sex were: (1) having no steady lover [for males: odds ratio (OR), 1.96, 95% confidence interval (CI), 1.41-2.70; for females: OR, 2.65; 95%CI, 1.56-4.84]; (2) younger age at the first sexual intercourse (for males: OR, 1.62, 95% CI, 1.22-2.15; for females: OR, 2.57; 95% CI, 1.64-4.02); (3) lack of condom use at the first sex (for males: OR, 1.13, 95% CI, 1.09-1.37; for females: OR, 2.81; 95% CI, 1.81-4.39); (4) unaware of the conditions of conception (for males: OR, 1.69, 95% CI, 1.31-2.19; for females: OR, 1.75; 95% CI, 1.16-2.65); and (5) unaware that abortion endangers women's future pregnancy (for males: OR, 2.98, 95% CI, 2.15-4.14; for females: OR, 2.34; 95% CI, 1.23-4.46). Medical graduates were not less likely to have unintended pregnancy than nonmedical graduates for both genders.

**Conclusions:**

The avoidable risk of being unintended pregnancy among graduate students in China indicates that an urgent need to take action on how to delay the age of first sex, promote condom use at first sex, and acquire accurate contraceptive information, as well as improve skills to use reliable contraception among graduate students.

## Background

Since China has been committed to the reform and opening-up policy to the world, premarital and extramarital sexual intercourse have been more common among young people in China [1-5]. With the growing incidence of premarital and extramarital sex in young people, unintended pregnancy and abortion have become seriously social and health issues in the country [6-8]. Due to China traditional idea of chastity and shortage of educational resources, Chinese young people, in contrast to their western counterparts, do not receive sufficient education of contraception, which often leads to misconceptions with reproduction and contraception [9,10].

China’s one child policy was introduced in 1979. It has been practiced to raise living standards by controlling family size and slowing down population growth. It stipulates that a couple should have only one child [11]. As a result, a largest National Family Planning Program in the world has been established in China, and its primary responsibility is to provide family planning services, including contraceptive education, abortion, long-acting contraception (e.g. intrauterine contraceptive device (IUD), vasectomy and tubal ligation), and short-acting contraception (e.g. condom, contraceptives) [12]. But some reports showed that services of contraceptive education are still in shortage and that short-acting contraception suitable for persons who have no child are mainly aimed at married persons or at women that are going to have premarital examinations [7,13,14]. Therefore, many unmarried young people did not know where or how to obtain contraception 10 years ago [15]. And, as yet some unmarried youth do not know what are reliable contraception, how to access and use reliable and free contraception [16,17]. Furthermore, lack of counseling and privacy in these services prevents many unmarried people from seeking contraception services [5].

Up to now, China has more than 1.4 million graduate students [18]. Most graduate students are over the legal age for marriage. However, few graduates in China choose to marry their lovers during studies due to the pressure of employment and learning [19]. As a result, they have a longer period between their initiation of sexual activity and marriage than their out-of-school counterparts [19]. On the other hand, it is believed that people with higher education know more contraceptive knowledge [20-22]. Whether the delay of marriage can result in the increase of premarital sex among graduate students, whether they can prevent themselves from unintended pregnancy and abortion more effectively, and whether family planning services can meet their need remain to be clarified.

A section-crossing study was conducted on premarital sexual intercourse and unintended pregnancy among graduate students in different regions from 2007 to 2008. The goal is to ascertain the prevalence rate of premarital sex and unintended pregnancy, to assess possible risk factors of pregnancy for graduate students active in premarital sex, and to obtain background information for further family planning services and contraceptive education.

## Methods

### Study setting

From September 15, 2007 to January 15, 2008, a cross-sectional descriptive study was conducted in 49 colleges/universities in 7 cities of China, including 12 universities in North China (6 in Beijing and 6 in Changchun), 16 in West China (8 in Chongqing and 8 in Chengdu), 7 in East China (7 in Nanjing), 7 in South China (7 in Nanning), and 7 in Central China (7 in Wuhan).

### Sampling

A multistage, stratified, cluster sampling was recruited. Firstly, purposive sampling was used to select city from North, West, East, South and Central of China, respectively. Then, stratified random sampling was used to choose college/university: (a) universities/colleges were divided into four layers (comprehensive, science and engineering, literature and history, and medical); (b) two universities/colleges were randomly sampled from each layer(all schools were selected if the number of schools of this layer less than or equal to 2). In the third stage, stratified cluster sampling was used to select class: (a) classes were divided into two layers (doctoral candidate and master candidate); (b) six classes of master candidate were randomly sampled from university/college that only has master's degree authorization; six classes of master candidate and two classes of doctoral candidate were randomly sampled from university/college that have both master's degree and doctoral’ degree authorization); (c) all unmarried graduate students (the marriage status was recorded in school) in these chosen classes were recruited, which based on ethical guidelines.

As a result, 13,544 voluntary participants were recruited from 327 classes of 49 universities/colleges of 7 cities. Among them, 88.1% (11,936) responded validly and completely. Only those who met the description of the corresponding questions were included in analysis. The sample of graduate students active in premarital sex was composed of 1831 males and 1059 females.

### Ethics issue

The topic was an observational study. The ethical issues of Informed Consent and Privacy were fully considered (all students from particular classes were informed by their instructors for the aim of the survey, and students’ participation was voluntary and confidential. Each participant signed the informed consent form before the survey. The self-administered questionnaires were distributed among students in classroom with members of the research team present only at the completion of the questionnaire. All answer sheets were made anonymous and tagged by arbitrary codes). The ethics committee of *Institute of Family Planning, Tongji Medical College, Huazhong University of Science and Technology* reviewed, and voted to approve the study (No. 2007–012).

### Questionnaire survey and data collection

The self-completed questionnaire was designed after a thorough review of similarly published reports. Pilot tests on a group of 800 undergraduates from various grades, disciplines, both genders were randomly selected in Wuhan to determine whether the concepts and expressions used in the questionnaire could be easily comprehended. The modified questionnaire consisted of an introduction, personal basic information (gender, age, educational level, discipline and whether having a steady lover), and 48 items covering three topics: (1) awareness of contraception, pregnancy and abortion; (2) attitudes towards abortion; and (3) practices of contraception, pregnancy and abortion. The self-administered questionnaires were distributed among students in classroom then collected *locus in quo* in 30 minutes.

### Data analysis

Questionnaire data was proofread and entered in EPIdata (version 3.02), and was analyzed using Statistical Package for Social Sciences (SPSS version 12.0). Factors associated with pregnancy among female graduate students/male students’ partners were determined in univariate analyses, and were categorized and listed below: (1) basic personal information (educational levels, disciplines, whether having a steady lover, disposable income per month and age at the first sexual intercourse); (2) awareness of contraception (whether knowing the conditions of conception, whether knowing the use of the menstrual cycle for contraception); (3) attitudes towards abortion (whether approving that abortion endangers women's physical and mental health, and future pregnancy); and (4) practices of contraception (whether using contraceptives at the first sexual intercourse, Whether use of condoms at first sexual intercourse, and frequency of use of contraception).

Variables significant in the univariate analyses (p<0.05) were then entered into a multivariate logistic regression model using forward stepwise logistic regression.

## Results

### Characteristics of the study participants

A total of 11,936 graduate students provided valid answer sheets that supplied details of gender, discipline, and contained responses to more than 90% of the questions. Among them, 49.4% was male; 21.9% was medical student; 12.5% was doctoral candidate; and up to 70.1% of responders reported having a steady lover. Their average disposable income per month was 727.2 Yuan; and their average age was 29.4 years (Table 1).

**Table 1:** Sociodemographic characteristics of unmarried graduate students

	**Male(n=5900)**		**Female(n=6036)**		**Total(n=11936)**	
	**n**	**%**	**n**	**%**	**n**	**%**
Educational level						
Doctoral candidate	852	14.4	641	10.6	1493	12.5
Master candidate	5048	85.6	5395	89.4	10443	87.5
Discipline						
Medical	1255	21.3	1357	22.5	2612	21.9
Nonmedical	4645	78.7	4679	77.5	9324	78.1
Having a stead lover						
Yes	4130	70.0	4235	70.2	8365	70.1
No	1770	30.0	1801	29.8	3571	29.9
Disposable income per month(Yuan, $\bar{x}\pm s$)	734.0±372.8		720.6±344.6		727.2±358.8	
Age(year, $\bar{x}\pm s$)	29.6 ±3.9		29.2±4.2		29.4±4.1	

### Prevalence of pregnancy and abortion

A history with unintended pregnancy was reported by 3.1% of females (17.9% (190/1059) of females active in premarital sex). Having a partner with pregnancy history was reported by 6.5% of males (20.8% (381/1831) of males active in premarital sex). A history with abortion was reported by 3.1% of females (97.9% (186/190) of pregnant females). Having a partner with abortion history was reported by 6.2% of males (96.1% (366/381) of males having partners with pregnancy history) (Table 2).

**Table 2:** Practice for unintended pregnancy among unmarried graduate students

	**Male(n=5900)**		**Female(n=6036)**		**Total(n=11936)**	
	**n**	**%**	**n**	**%**	**n**	**%**
History of premarital sexual intercourse (have you experienced at least one times premarital sexual intercourse?)						
Yes	1831	31.0	1059	17.5	2890	24.2
No	4069	69.0	4977	82.5	9046	75.8
History of unintended pregnancy(have(has) you(your partner) experienced at least one times pregnancy?)						
Yes	381	6.5	190	3.1	571	4.8
No	5519	93.5	5846	96.9	11365	95.2
History of abortion(have(has) you(your partner) experienced at least one times abortion?)						
Yes	366	6.2	186	3.1	552	4.6
No	5534	93.8	5850	96.9	11384	95.4

### Characteristics of students active in premarital sex

Among the students active in premarital sex, 88.3% of males and 92.4% of females reported that they had a steady lover. 18.9% of males and 11.1% of females were less than 20 years old at their first sexual intercourse. 75.6% of males and 80.4% of females knew the conditions of conception. 59.4% of males and 75.6% of females knew the use of menstrual cycle for contraception. 79.3% of males and 85.5% of females agreed that abortion endangers women's physical and mental health. 89.8% of males and 95.1% of females agreed that abortion endangers women’s future pregnancy. 36.9% of males and 30.4% of females used condoms in their first sexual intercourse. 29.9% of males and 33.8% of females always used contraception in sexual intercourses (Table 3).

**Table 3:** Factors for unintended pregnancy among graduate students active in premarital sex

**Factors**	**Male students (n=1831)**			**Female students (n=1059)**		
	**Total n (%)**	**IOP**^**a **^**n (%)**	**COR**^**b**^**(95% CI)**	**Total n (%)**	**IOP**^**a **^**n (%)**	**COR**^**b**^**(95% CI)**
Educational levels						
Master candidate	1467(80.1)	296(20.2)	1	885(83.6)	148(16.7)	1
Doctoral candidate	364(19.9)	77(21.2)	1.06(0.80,1.41)	174(16.4)	40(23.0)	1.49(1.01,2.22)
Discipline						
nonmedical	1434(78.3)	299(20.9)	1	818(77.2)	137(16.7)	1
medical	397(21.7)	74(18.6)	0.87(0.66,1.15)	241(22.8)	51(21.2)	1.33(0.93,1.91)
Whether having a steady lover						
Yes	1616(88.3)	302(18.7)	1	979(92.4)	156(15.9)	1
No	215(11.7)	71(33.0)	2.13(1.56,2.94)	80(7.6)	32(40.0)	3.57(2.17,5.56)
Disposable income per month						
<400(yuan)	360(19.7)	79(21.9)	1.13(0.86,1.58)	184(17.4)	47(25.5)	1.90(1.26,2.87)
400~899 (yuan)	792(43.3)	154(19.4)	1	503(47.5)	77(15.3)	1
≥900(yuan)	679(37.1)	140(20.6)	1.08(0.83,1.39)	372(35.1)	64(17.2)	1.15(0.80,1.65)
Age at first sexual intercourse						
<20 years	346(18.9)	91(26.3)	1.53(1.16,2.00)	118(11.1)	39(33.1)	2.62(1.72,4.00)
≥20 years	1485(81.1)	282(19.0)	1	941(88.9)	149(15.8)	1
Whether knowing the conditions of conception						
Yes^c^	1384(75.6)	243(17.6)	1	851(80.4)	134(15.7)	1
No	447(24.4)	130(29.1)	1.93(1.50,2.46)	208(19.6)	54(26.0)	1.88(1.31,2.69)
Whether knowing the use of the menstrual cycle for contraception						
Yes^d^	1087(59.4)	199(18.3)	1	801(75.6)	130(16.2)	1
No	744(40.6)	174(23.4)	1.36(1.08,1.71)	258(24.4)	58(22.5)	1.50(1.06,2.12)
Whether thinking abortion endanger women's physical and mental health						
Yes	1452(79.3)	280(19.3)	1	905(85.5)	148(16.4)	1
No	379(20.7)	93(24.5)	1.36(1.04,1.78)	154(14.5)	40(26.0)	1.80(1.20,2.68)
Whether thinking abortion endanger women’s future pregnancy						
Yes	1645(89.8)	293(17.8)	1	1007(95.1)	166(16.5)	1
No	186(10.2)	80(43.0)	3.48(2.54,4.78)	52(4.9)	22(42.3)	3.72(2.09,6.62)
Whether use of condoms at first sexual intercourse						
Yes	675(36.9)	119(17.6)	1	322(30.4)	27(8.4)	1
No	1156(63.1)	254(22.0)	1.24(1.02,1.51)	737(69.6)	161(21.8)	3.03(2.00,4.76)
Whether use of contraceptives at first sexual intercourse						
Yes	1718(93.8)	341(19.8)	1	964(91.0)	168(17.4)	1
No	113(6.2)	32(28.3)	1.60(0.94,2.44)	95(9.0)	20(21.1)	1.26(0.75,2.13)
Frequency of use of contraception(methods were looked as effective for preventing pregnancy by responders)						
Always use	548(29.9)	105(19.2)	1.71(0.83,3.55)	358(33.8)	48(13.4)	0.40(0.16,0.91)
Often/sometimes	1209(66.0)	259(21.4)	1.97(0.97,4.01)	676(63.8)	133(19.7)	0.63(0.26,1.53)
Never use	74(4.1)	9(12.2)	1	25(2.4)	7(28.0)	1

^a^: incidence of pregnancy; ^b^: crude odds ratio; ^c^: students select all correct answers of conditions of conception but no wrong answers from list [detail of information was showed as below: *What are the basic conditions for human conception? (Multiple choice) 1, healthy and active sperm can get into the vagina, and remain active force; 2, healthy and mature egg; 3, cervical mucus must be appropriate and conducive to sperm survival and penetration; 4, unobstructed reproductive tract to make the sperm and egg meeting; 5, sperm and egg must combined and formed a fertilized egg; 6, fertilized egg reach the uterine cavity timely, and the intrauterine environment is suitable for the growth and development of fertilized egg. 7, the sex intercourse between male and female.* If a student chose answer 1,2,3,4,5 and 6 but no 7,.we considered it as ‘knowing the conditions of conception’, otherwise ‘no’]; ^d^: students select right answers of calculating periodic abstinence and select the answer of periodic abstinence being not completely safe for contraception from list.

### Univariate correlation of pregnancy among sexually active students

In the univariate analyses, (1) having no steady lover (VS. yes), (2) initiating sex before 20 years old (vs. initiating after 20 years old), (3) unawareness of the conditions of conception (VS. awareness), (4) no using condoms in the first sexual intercourse (VS. using), (5) unawareness of the menstrual cycle for contraception (VS. awareness), (6) unawareness of that abortion endangers women's physical and mental health (VS. awareness), and (7) unawareness of that abortion endangers women’s future pregnancy (VS. awareness) were related to unintended pregnancy among both females and males’ partners. For female graduates, being doctoral candidate (VS. being master candidate), having disposable income less than 400 Yuan per month (VS. having disposable income between 400 and 900 Yuan per month), and no using contraception in their sexual intercourse (VS. always using) suggest more likelihood of reporting unintended pregnancy. Medical graduates were not less likely to have unintended pregnancy than nonmedical graduates for both genders (Table 3).

### Multivariate correlation of pregnancy among sexually active students

In the multivariate logistic regression analysis, (1) having no steady lover [male: odds ratio (OR), 1.96; 95% confidence interval (CI), 1.41-2.70. female: OR, 2.65; 95% CI, 1.56-4.84], (2) younger age in the first sexual intercourse (male: OR, 1.62; 95% CI, 1.22-2.15. female: OR, 2.57; 95% CI, 1.64-4.02), (3) lack of condom use in the first sex (male: OR, 1.13; 95% CI, 1.09-1.37. female: OR, 2.81; 95% CI, 1.81-4.39), (4) unawareness of the conditions of conception (male: OR, 1.69; 95% CI, 1.31-2.19. female: OR, 1.75; 95% CI, 1.16-2.65), and (5) unawareness of that abortion endangers women’s future pregnancy (male: OR, 2.98; 95% CI, 2.15-4.14. female: OR, 2.34; 95% CI, 1.23-4.46) were the risk factors for unintended pregnancy for both genders. (Table 4).

**Table 4:** Factors associated with unintended pregnancy among graduate students active in premarital sex

**Factors**	**Male students (n=1831)**	**Female students (n=1059)**
	**AOR**^**a**^**(95%CI)**	**AOR**^**a**^**(95%CI)**
Whether having steady lover		
Yes	1	1
No	1.96(1.41,2.70)	2.65(1.56,4.84)
Age in first sexual intercourse		
<20 years	1.62(1.22,2.15)	2.57(1.64,4.02)
≥20 years	1	1
Whether using condoms in first sexual intercourse		
Yes	1	1
No	1.13(1.09,1.37)	2.81(1.81,4.39)
Whether knowing the conditions of conception		
Yes	1	1
No	1.69(1.31,2.19)	1.75(1.16,2.65)
Whether thinking abortion endanger women’s future pregnancy?		
Yes	1	1
No	2.98(2.15,4.14)	2.34(1.23,4.46)

AOR, adjusted odds ratio.

## Discussion

In China, reproductive health care is widely available through National Family Planning services. In the last three decades, China has experienced dramatically social changes associated with rapid economic growth and reform. Traditional attitudes towards unmarried cohabitation and premarital sex have also been changed quickly [9,13,23]. More young adults with sexual relationship are not legally married. However, social responses to these changes are slow [7]. The current Family Planning Programme still targets mainly on married couples rather than unmarried young people [24].

We found unmarried graduates’ average age was 29.4. It was much higher than the legal age for marriage (≥22 for male and ≥20 for female) in China. During the long period between their initiation of sexual activity and marriage, 31.0% of male graduates and 17.5% of female graduates reported a history of premarital sexual intercourse, which was higher than those among undergraduate students (males: 15.4%~20.2%; females: 9.4%~10.6%) [2,4,8] and among their out-of school counterparts (males: 20.2%~21.4%; females: 7.1%~8.7%) [14,25]. According to the data, 70.0% of males and 70.2% of females had a steady lover. We believed that the real prevalence rate of premarital sexual intercourse was much higher than reported. It seemed that unmarried graduate students also need the family planning services.

The prevalence rate of unintended pregnancy among graduate students active in premarital sex was 19.8% (571/2890). It was lower than undergraduate students (23.1%~31.5%) [8,26,27] and their out-of school counterparts(50%~64.2%) [14,28], which might indicate graduate students could prevent themselves from unintended pregnancy more effectively. But we also found nearly all pregnant graduates (96.7%, 552/571) chose abortion to end pregnancy, which was much higher than undergraduates (78.7%) [13] and their out-of school counterparts(25%) [6]. The possible reasons for this might be as fellows: (*a*) they had no money to feed a baby. Most of them could only get poor living allowances from schools to feed themselves (graduates’ disposable income per month was 727 Yuan; Chinese Per Capita Month Disposable Income of Urban Households was 1315 Yuan and Engel's Coefficient of Urban Households was 37.9% in 2008 [29]); (*b*) they had no time to feed a baby. Most of them have to spend all their time studying or looking for jobs. In contrast, their out-of-school counterparts who had premarital pregnancy chose to have prompt marriage in most cases [6]; (*c*) the sexual relationship was not stable for some graduate students. From these, we believed that the convenient family planning services for graduate students was in demand.

We found students with a steady lover had a significantly lower rate of unintended pregnancy. It might indicate that students with a steady lover were much better in preparation for contraception. In contrast, “one-night-stand” students without a steady lover had difficulty in preparing contraception timely due to the risk of being stigmatized for their searching of contraceptive services [5]. Hence, we believed that convenient contraceptive services offer for them might effectively decrease the rate of unintended pregnancy.

Students having sex at younger ages were more likely to report a history of unintended pregnancy in both genders in our study. We presumed that two factors might be important. The first was that students who initiated sex at early age were lacking in awareness of the risks of pregnancy and utilized contraception less often than late initiators did [30]. The second was that the students with earlier initiation of sexual intercourse would be exposed to the longer periods of the risk of unintended pregnancies [24].

Our data showed both genders that used condom in the first sex had a significantly lower rate of unintended pregnancy. It might be that those who used condoms in the first sex has increased likelihood of subsequent condom use [31]. It was regretted that the rate of condom use in the first sex was only 30.4% for females' partners and 36.9% for males. Condoms, OCs, withdrawal and periodic abstinence were considered to be the most common contraceptive methods among unmarried people [6,8]. However, the last two methods often led to contraceptive failure [32]. We found that 29.9% of male graduates and 33.8% of female graduates always used contraception (methods were looked as effective for preventing pregnancy by responders) in their sexual intercourse. But the behavior could not effectively prevent them from unintended pregnancy. We also noticed that OR for female was <1, but OR for male was >1 of being pregnant (always use contraception that were looked as effective for preventing pregnancy by responders *VS* never use). We presumed that one of possible reasons was that self-reported ‘use of contraception' was not necessarily reliable method. Another possible factor was that male students had more possible classify experience of unintended pregnancy to contraceptive failure instead of absence of contraception. All of these indicates that how to select and how to use contraception correctly might still confuse graduate students.

Unawareness of the conditions of conception and unawareness of that abortion endangers women’s future pregnancy were related to pregnancy for both genders. It indicated that improving the awareness of reproduction and contraception might decrease the prevalence rate of unintended pregnancy among graduate students.

Our current data unexpectedly showed that medical students were not less likely to have unintended pregnancy than nonmedical students. In China, nearly all school and college do not set up reproductive and contraceptive courses. And due to the constrained Chinese traditional concept of chastity, students can not obtain correct contraceptive knowledge from society or family. We presumed that medical graduates might know more about the function and structure of the reproductive system than nonmedical graduates, but still lacked the knowledge of how to prevent unintended pregnancy.

Based on the multistage, stratified, cluster design, our sample came from diverse regions of China involving various disciplines and university levels. It could reduce the selection bias and nondifferential misclassification in the greatest possible way. Therefore, we believed our findings could be generalized. However, our study might have some bias: (a) for the sensitive question of premarital sex, no special technique (such as randomized responses) was used in the study, which might lead to underestimated rate of premarital sex; (b) for those male students who were reported unintended pregnancy for their partners, we could not judge whether the unintended pregnancy was caused by themselves or not, particularly for those who did not mention a steady lover; and (c) cause-effect relation could not be established and recall bias could not be avoided due to the cross-sectional nature of the study.

## Conclusions

The contraceptive education and family planning services supplied in China are lagging behind the current demands of graduate students; the prevalence rate of unintended pregnancy and abortion among graduate students active in premarital sex is high; and some risk of being unintended pregnancy is avoidable. Therefore, as the main contraceptive education and services provider, the family planning service system is in an urgent need to take action on how to delay the age of first sex, promote condom use at first sex, and acquire accurate contraceptive information, as well as improve skills to use reliable contraception among graduate students.

## Competing interests

The authors declare that they have no competing interests.

## Authors' contributions

CLX, XJS, GHL, MMZ and PY designed the survey. YZZ, JWX, XJS, GHL, MMZ, PY and CLX contributed by performing the investigation and data collection. YZZ and JWX performed the statistical analysis and articles written. All authors read and approved the final manuscript.

## Pre-publication history

The pre-publication history for this paper can be accessed here:

http://www.biomedcentral.com/1471-2458/13/198/prepub

## References

[B1] Yan H Li L Bi Y Xu X Li S Maddock JE Family and peer influences on sexual behavior among female college students in Wuhan, China Women Health 2010 50 8 767 782 10.1080/03630242.2010.530926 21170818

[B2] Zhang L Gao X Dong Z Tan Y Wu Z Premarital sexual activities among students in a university in Beijing, China Sex Transm Dis 2002 29 4 212 215 10.1097/00007435-200204000-00005 11912462

[B3] Cao Y Zhou X Wang X He Q Lui Z Yang Y Ji Y Sexual knowledge, behaviors, and attitudes of medical students in Kunming, China Psychol Rep 1998 82 1 201 202 9520554 10.2466/pr0.1998.82.1.201

[B4] Chen B Lu Y Wang H Ma Q Zhao X Guo J Hu K Wang Y Huang Y Chen P Sexual and reproductive health service needs of university/college students: updates from a survey in Shanghai, China Asian journal of andrology 2008 10 4 607 615 10.1111/j.1745-7262.2008.00413.x 18478161

[B5] Zhang L Jejeebhoy S Shah IH Zhang L Hsia J Im-em W Access to contraceptive services among unmarried young people in the north-east of China Eur J Contracept Reprod Health Care 2004 9 3 147 154 10.1080/13625180400007181 15697104

[B6] Che Y Cleland J Contraceptive use before and after marriage in Shanghai Stud Fam Plann 2003 34 1 44 52 10.1111/j.1728-4465.2003.00044.x 12772445

[B7] Qian X Tang S Garner P Unintended pregnancy and induced abortion among unmarried women in China: a systematic review BMC Health Serv Res 2004 4 1 1 10.1186/1472-6963-4-1 14736336 PMC333425

[B8] Zhou Y Zhang M Wei S Guan H Yin P Ren N Pang X Xiong C Survey on knowledge, attitude, practice related to contraception among college students in Beijing Zhonghua Liu Xing Bing Xue Za Zhi 2009 30 7 710 712 19957597

[B9] Huang J Bova C Fennie KP Rogers A Williams AB Knowledge, attitudes, behaviors, and perceptions of risk related to HIV/AIDS among Chinese university students in Hunan, China AIDS Patient Care STDS 2005 19 11 769 777 10.1089/apc.2005.19.769 16283837

[B10] WHO, UNICEF A picture of health: A review and annotated bibliography of the health of young people in developing countries 1995 Geneva: WHO

[B11] Torjesen I Chinese would resist having larger families if one child policy was relaxed BMJ 2010 340 c1212 10.1136/bmj.c1212 20200059

[B12] Family Planning Technical Service Management Ordinance http://www.gov.cn/zwgk/2005-05/23/content_262.htm]

[B13] Zhou Y Xiong C Yin P Shang X Wei S Liu H Liu G Wang X Zhang M Li X Guan H Xiao D Pang X Ren N Wang M Survey of Status and Requirement about Sexual Behavior and Contraception among Unmarried College/University Students in China Acta Med Univ Sci Technol Huazhong 2009 38 5 561-570 580

[B14] Xiong J Yin P Guan H Pang X Xiong C Analysis on contraception status and service requirements of high- income youth in Wuhan Chinese Journal of Maternal and Child Care 2010 25 6 785 789

[B15] Zheng Z Zhou Y Zheng L Yang Y Zhao D Lou C Zhao S Sexual behaviour and contraceptive use among unmarried, young women migrant workers in five cities in China Reprod Health Matters 2001 9 17 118 127 10.1016/S0968-8080(01)90015-1 11468827

[B16] Chen H Zhang L Han Y Lin T Song X Chen G Zheng X HIV/AIDS knowledge, contraceptive knowledge, and condom use among unmarried youth in China AIDS Care 2012 24 12 1550 1558 10.1080/09540121.2012.674093 22533677

[B17] Liu Z Zhu M Dib HH Li Z Shi S Wang Z RH knowledge and service utilization among unmarried rural-to-urban migrants in three major cities China BMC Publ Health 2011 11 74 10.1186/1471-2458-11-74 PMC3044659 21284893

[B18] Chinese Ministry of Education Chinese Education Statistics in 2009 2010 http://www.moe.edu.cn/publicfiles/business/htmlfiles/moe/s4960/201012/113589.html

[B19] Zhou X On the problem of marriage for the youth with a high educational background: A report on the marriage and the concept of child bearing of the Chinese youth Journal of China Youth College for Political Sciences 2002 21 4 21 26

[B20] Wang L Yu J Li X Fan Y Tao G Zhang H Analysis of contraceptive knowledge level and influencing factors among reproductive women in Shandong province Chinese Journal of Maternal and Child Care 2007 22 8 1096 1099

[B21] Yin X Analysis of contraceptive knowledge level and influencing factors among unmarried women Chinese Journal of Maternal and Child Care 2005 19 1 55 56

[B22] Zhou Y Han T Huang L Knowledge, attitude, and behavior to contraception among child-bearing women Journal of Nursing Science 2010 25 4 27 29

[B23] Zhang K Li D Li H Beck EJ Changing sexual attitudes and behaviour in China: implications for the spread of HIV and other sexually transmitted diseases AIDS care 1999 11 5 581 589 10.1080/09540129947730 10755033

[B24] Ma Q Ono-Kihara M Cong L Xu G Pan X Zamani S Ravari SM Kihara M Unintended pregnancy and its risk factors among university students in eastern China Contraception 2008 77 2 108 113 10.1016/j.contraception.2007.10.008 18226674

[B25] Zhao F Wang L Guo S Wu J Ye S Investigation on sexual behavior with non-spouse and condom use among reproductive age men and women Chin J Publ ic Heal th 2006 22 11 1309 1310

[B26] Zhou Y Xiong J Li J Huang S Shang X Liu G Zhang M Yin P Wei S Xiong C Urgent Need for Contraceptive Education and Services in Chinese Unmarried Undergraduates: A Multi-campus Survey J Huazhong Univ Sci Technol[Med Sci] 2011 31 3) 342 347 10.1007/s11596-011-0468-2 21823000

[B27] Song Y Ji CY Sexual intercourse and high-risk sexual behaviours among a national sample of urban adolescents in China J Public Health (Oxf) 2010 32 3 312 321 10.1093/pubmed/fdp123 20147385

[B28] Ye J Huang S Ao G Premarital sex knowledge and sexual behaviors survey to unmarried working male youths The Chinese Journal of Human Sexuality 2010 19 8 32–34 40

[B29] National Bureau of Statistics of China China Statistical Year book in 2009: Per Capita Annual Income and Engel's Coefficient of Urban and Rural Households 2009 [http://www.stats.gov.cn/tjsj/ndsj/2009/html/J0902e.htm]

[B30] Bender SS Kosunen E Teenage contraceptive use in Iceland: a gender perspective Public Health Nurs 2005 22 1 17 26 10.1111/j.0737-1209.2005.22104.x 15670321

[B31] Shafii T Stovel K Davis R Holmes K Is condom use habit forming? Condom use at sexual debut and subsequent condom use Sex Transm Dis 2004 31 6 366 372 10.1097/00007435-200406000-00010 15167648

[B32] Cheng Y Gno X Li Y Li S Qu A Kang B Repeat induced abortions and contraceptive practices among unmarried young women seeking an abortion in China Int J Gynaecol Obstet 2004 87 2 199 202 10.1016/j.ijgo.2004.06.010 15491580

